# Novel Prehospital Prediction Model of Large Vessel Occlusion Using Artificial Neural Network

**DOI:** 10.3389/fnagi.2018.00181

**Published:** 2018-06-26

**Authors:** Zhicai Chen, Ruiting Zhang, Feizhou Xu, Xiaoxian Gong, Feina Shi, Meixia Zhang, Min Lou

**Affiliations:** ^1^Department of Neurology, The Second Affiliated Hospital of Zhejiang University, School of Medicine, Hangzhou, China; ^2^Department of Engineering, Microcloud Communication Technology, Hangzhou, China

**Keywords:** large vessel occlusion, artificial neural network, stroke, scale, NIHSS

## Abstract

**Background**: Identifying large vessel occlusion (LVO) patients in the prehospital triage stage to avoid unnecessary and costly delays is important but still challenging. We aim to develop an artificial neural network (ANN) algorithm to predict LVO using prehospital accessible data including demographics, National Institutes of Health Stroke Scale (NIHSS) items and vascular risk factors.

**Methods**: Consecutive acute ischemic stroke patients who underwent CT angiography (CTA) or time of flight MR angiography (TOF-MRA) and received reperfusion therapy within 8 h from symptom onset were included. The diagnosis of LVO was defined as occlusion of the intracranial internal carotid artery (ICA), M1 and M2 segments of the middle cerebral artery (MCA) and basilar artery on CTA or TOF-MRA before treatment. Patients with and without LVO were randomly selected at a 1:1 ratio. The ANN model was developed using backpropagation algorithm, and 10-fold cross-validation was used to validate the model. The comparison of diagnostic parameters between the ANN model and previously established prehospital prediction scales were performed.

**Results**: Finally, 300 LVO and 300 non-LVO patients were randomly selected for the training and validation of the ANN model. The mean Youden index, sensitivity, specificity and accuracy of the ANN model based on the 10-fold cross-validation analysis were 0.640, 0.807, 0.833 and 0.820, respectively. The area under the curve (AUC), Youden index and accuracy of the ANN model were all higher than other prehospital prediction scales.

**Conclusions**: The ANN can be an effective tool for the recognition of LVO in the prehospital triage stage.

## Introduction

Endovascular thrombectomy (EVT) for treatment of acute large vessel occlusion (LVO) has been widely accepted around the world currently (Powers et al., 2015; Michel, 2017). Similar to intravenous thrombolysis, EVT is highly time dependent (Emberson et al., 2014; Sheth et al., 2015). However, hospitals with around-the-clock endovascular capability are scarce in many parts of the world, presenting a serious challenge to current systems of care for the organization and delivery of appropriate patients to endovascular-capable centers.

There are growing literatures about clinical scales that aim to differentiate LVO from milder strokes and thus allow paramedics to rapidly identify LVO in the prehospital setting, including Field Assessment Stroke Triage for Emergency Destination (FAST-ED; Lima et al., 2016), 3-item Stroke Scale (3I-SS; Singer et al., 2005), the Rapid Arterial Occlusion Evaluation Scale (RACE; Pérez de la Ossa et al., 2014), Prehospital Acute Stroke Severity scale (PASS; Hastrup et al., 2016), Cincinnati Prehospital Stroke Severity Scale (CPSSS; Katz et al., 2015) and Los Angeles Motor Scale (LAMS; Nazliel et al., 2008). These scales are mainly transformed from National Institutes of Health Stroke Scale (NIHSS) items, and the algorithm of these scales are all based on the hypothesis of linear correlation between input parameters and the presence of LVO. However, they ignore the intricate associations among the input parameters, and none of these scales concurrently incorporate other stroke-related parameters, such as age, medical history and vascular risk factors.

The artificial neural network (ANN), inspired by the neuronal networks in the central nervous system, is widely used for prediction and classification now. It consists of nodes that are connected together to form a network with variable strengths (weights) between different connections. Through training, the relationship between input parameters and outputs in the ANN can be mapped by altering the weights within the network. It is particularly effective at capturing nonlinear relationships that make ANN an ideal candidate for multifactorial disease classification. Recent studies also demonstrated that ANN is ideal for prediction of disease diagnosis such as acute ischemic stroke, acute coronary syndromes and cancers (Isma’eel et al., 2016; Abedi et al., 2017; Esteva et al., 2017).

Therefore, the aim of our study was to develop an ANN algorithm to identity LVO using prehospital accessible data including demographics, NIHSS items, medical history and vascular risk factors and then compare its diagnostic parameters with previously established prehospital prediction scales.

## Materials and Methods

### Study Population

We retrospectively reviewed our prospectively collected database (Zhang R. et al., 2017; Zhang S. et al., 2017) for consecutive acute ischemic stroke patients who underwent CT angiography (CTA) or time of flight MR angiography (TOF-MRA) and received reperfusion therapy within 8 h from symptom onset in our center from June 2009 to February 2017. Baseline clinical variables including age, gender, prior antiplatelet therapy, risk factors (smoking, hypertension, diabetes mellitus, hyperlipidemia, history of stroke/TIA, atrial fibrillation, hyperhomocystinemia, coronary artery disease and family history of cerebrovascular disease) and complete breakdown of baseline NIHSS (15 items) were collected by medical staff from patients and families in the emergency department. Uncertain histories were denoted as negative. LVO was defined as occlusion of the intracranial internal carotid artery (ICA), M1 and M2 segments of the middle cerebral artery (MCA) and basilar artery (Lima et al., 2016) on CTA or TOF-MRA before treatment.

### Ethics Statement

All subjects had given written informed consent prior to the study, and the protocols had been approved by the human ethics committee of the second affiliated hospital of Zhejiang University, School of Medicine. Clinical investigation had been conducted according to the principles expressed in the Declaration of Helsinki.

### Data Analysis

**Figure 1:**
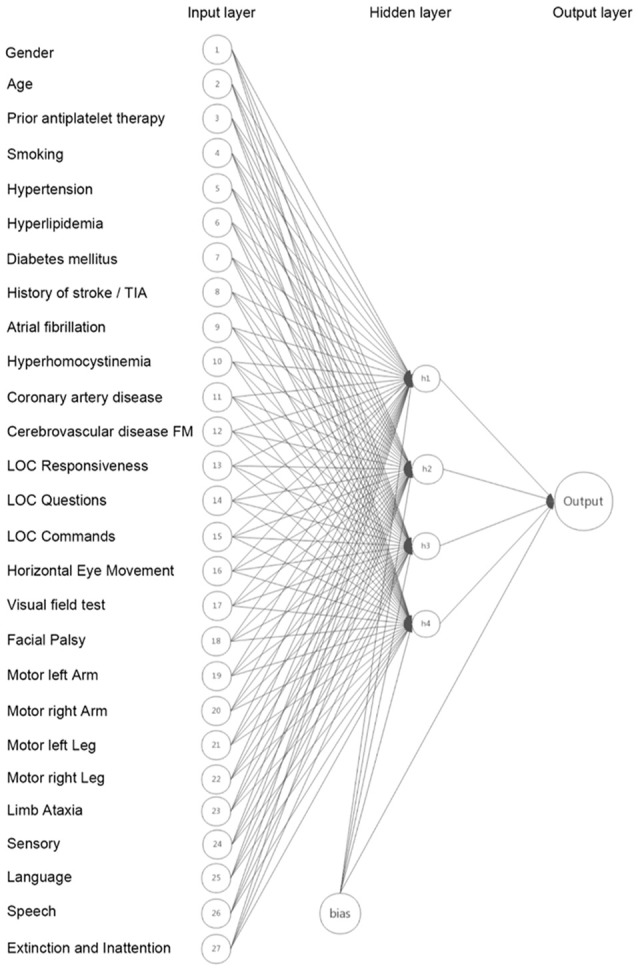
The Artificial neural network (ANN) model developed based on age, gender, prior antiplatelet therapy, 15 National Institutes of Health Stroke Scale (NIHSS) items and nine risk factors.

We developed the ANN model using Scikit-Learn package in python software (Venthur et al., 2015). In general, the ANN model consists of three layers: an input layer that receives information, a hidden layer that processes information, and an output layer that calculates results (Metgud et al., 2013). The input layer contained neurons of age, gender, prior antiplatelet therapy, 15 NIHSS items and nine risk factors selected according to published literatures and pathophysiologic consideration. The hidden layer consisted of four neurons which was optimized with a different number of hidden neurons. The output layer consisted of one neuron. Figure 1 represented the ANN model development. The backpropagation algorithm was used to minimize the error function (Abedi et al., 2017). Backpropagation is a method to calculate the gradient of the loss function with respect to the weights in an ANN. It is commonly used as a part of algorithms that optimize the performance of the network by adjusting the weights. The number of LVO and non-LVO patients was 1:1 matched to ensure a balanced data set to make sure that the larger group was not over-represented in the data set. Randomized identification number from a uniform distribution was randomly assigned to each patient, and patients were selected from the lowest identification number to create the full balanced data set. Supplementary Figure S1 summarizes the study selection process. We performed 10-fold cross-validation to assess the generalizability of our model (Abedi et al., 2017). In 10-fold cross-validation, the original sample was randomly partitioned into 10 equal sized subsamples by programming computer with instructions. Of the 10 subsamples, a single subsample was retained as the validation data to test the model, and the remaining nine subsamples were used as the training data. Each subsample consisted of 30 randomly selected LVO patients and 30 randomly selected non-LVO patients.

We then compared the diagnostic parameters of the ANN models with previously established prehospital prediction scales including FAST-ED, 3I-SS, RACE, PASS, CPSSS, LAMS, NIHSS, and NIHSS ≥ 6 in our database. To investigate whether stroke-related parameters, other than NIHSS can enhance the discrimination capacity, we also tested another ANN model including only 15 items of NIHSS (ANN model*).

### Statistical Analysis

Continuous variables were presented as the mean ± SD (normal distribution) and as median (IQR; skewed distribution). Receiver operating characteristic (ROC) analysis was used to get the area under the curve (AUC) of each prehospital prediction scale. The ROC derived optimal cutoff was determined at the maximal Youden Index. Finally, we calculated sensitivity, specificity and accuracy for the prediction of LVO.

## Results

A total of 777 patients were included (median age, 69 (IQR, 60–78) years; 483 males) in analysis. Median baseline NIHSS score was 10 (IQR, 4–15). Among them, 453 (58.3%) patients had LVO and 324 (41.7%) patients were in non-LVO group. Since we used 10-fold cross-validation to assess the generalizability of our model, which required that the sample could be divided by 10 exactly. Besides, the number of LVO and non-LVO patients was 1:1 matched to ensure a balanced data set to make sure that the larger group was not over-represented in the data set. Therefore, finally 300 LVO and 300 non-LVO patients were randomly selected from 777 patients for the training and validation of the ANN model.

Baseline demographics, medical history, NIHSS and risk factors were listed in Supplementary Table S1. The LVO patients were older (69 ± 13 vs. 66 ± 12, *p* < 0.001), had higher NIHSS (13 (8–17) vs. 6 (3–10), *p* < 0.001) and higher rate of atrial fibrillation (54.0% vs. 23.3%, *p* < 0.001) in comparison with non-LVO patients. There were no significant differences in other baseline variables between LVO and non-LVO groups.

**Figure 2:**
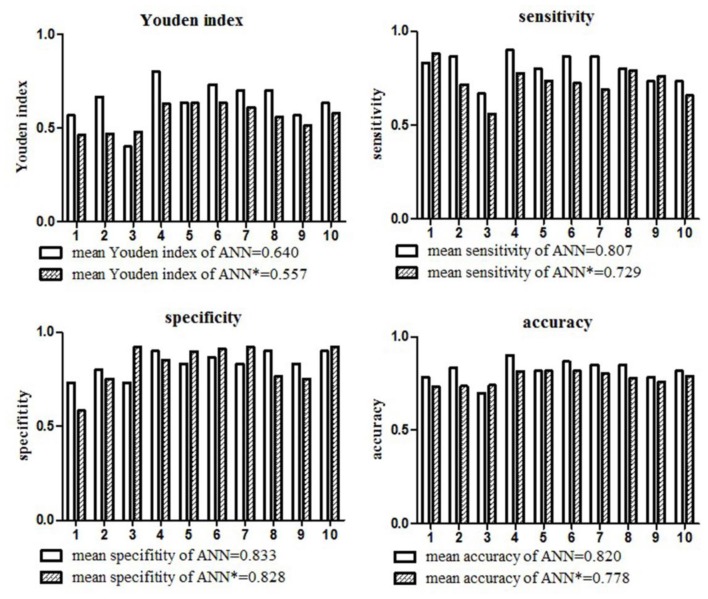
Diagnostic parameters of the ANN model in the 10 testing datasets. The mean Youden index, sensitivity, specificity and accuracy of the ANN model for the diagnosis of large vessel occlusion (LVO) were 0.640, 0.807, 0.833, and 0.820, respectively. The mean Youden index, sensitivity, specificity and accuracy of the ANN model* only included NIHSS items were 0.557, 0.729, 0.828, 0.778, respectively.

The mean Youden index, sensitivity, specificity and accuracy of the ANN model for the diagnosis of LVO based on the 10-fold cross-validation analysis were 0.640, 0.807, 0.833 and 0.820, respectively. The detailed diagnostic parameters of the ANN model in the 10 testing datasets were shown in Figure 2.

**Figure 3:**
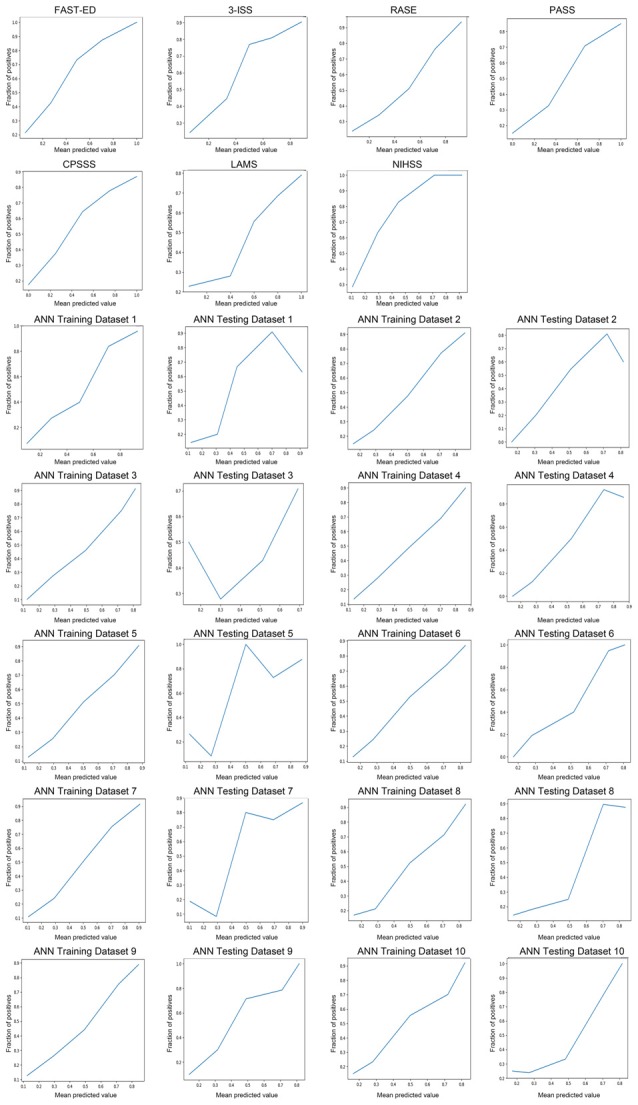
The calibration curves of predictions by ANN and other previously established prehospital prediction scales.

Table 1 showed the comparison of these diagnostic parameters between ANN model and previously established prehospital prediction scales including FAST-ED, 3I-SS, RACE, PASS, CPSSS, LAMS, NIHSS and NIHSS ≥6. The AUC, Youden index and accuracy of the ANN model ANN model* which included only NIHSS items were all better than other prehospital prediction scales. The calibration curves of predictions by ANN and other previously established prehospital prediction scales were shown in Figure 3.

**Table 1:** The comparison of diagnostic parameters between artificial neural network (ANN) model and previously established prehospital prediction scales.

	FAST-ED	3-ISS	RACE	PASS	CPSSS	LAMS	NIHSS	NIHSS ≥ 6	ANN	ANN*
AUC	0.783	0.782	0.776	0.784	0.796	0.740	0.790	/	0.823 ± 0.060	0.804 ± 0.042
Youden index	0.467	0.453	0.427	0.493	0.490	0.403	0.453	0.327	0.640 ± 0.105	0.557 ± 0.067
Sensitivity	0.760	0.583	0.730	0.727	0.713	0.807	0.607	0.847	0.807 ± 0.071	0.729 ± 0.081
Specificity	0.707	0.870	0.697	0.767	0.777	0.597	0.847	0.480	0.833 ± 0.060	0.828 ± 0.106
Accuracy	0.733	0.727	0.713	0.747	0.745	0.702	0.727	0.663	0.820 ± 0.053	0.778 ± 0.033
Cutoff	3	3	4	2	2	3	12	/	/	/

The ANN model included age, gender, prior antiplatelet therapy, 15 NIHSS items and nine risk factors, while ANN model* only included 15 NIHSS items. FAST-ED indicates Field Assessment Stroke for Emergency Destination, 3I-SS indicates 3-item Stroke Scale, RACE indicates the Rapid Arterial Occlusion Evaluation Scale, PASS indicates Prehospital Acute Stroke Severity scale, CPSSS indicates Cincinnati Prehospital Stroke Severity Scale and LAMS indicates Los Angeles Motor Scale.

## Discussion

To our knowledge, this was the first study that used ANN to predict acute LVO. Our results showed that the diagnostic parameters of ANN for the identification of LVO were higher than previously established prehospital prediction scales, which made it an effective tool for rapid recognition of LVO.

To date, no triage strategy performs perfectly in identifying LVO in the prehospital setting and it looks like we have to accept a certain proportion of over-diagnosis and under-diagnosis with any prehospital criteria, as we have already done with the current prehospital selection processes for intravenous thrombolysis (Michel, 2017). According to the current American Heart Association/American Stroke Association guidelines, NIHSS ≥6 is recommended to select eligible patients for EVT (Powers et al., 2015). However, in clinical practice, some certain items of NIHSS and specified patterns of combined deficits may carry a high attributable risk of LVO, but not contribute equally to the sum of score (Scheitz et al., 2017). Furthermore, given that right hemispheric symptoms are underrepresented with NIHSS, patients with right LVO might be misdiagnosed when they present mild to moderate neurological deficits (Jeffery, 1989). The simple addition algorithm of previously established prediction scales is thus not enough to detect all possible interactions between predictor variables. The advantage of the ANN algorithm is that it fits complex nonlinear relationships between input parameters and outputs until reaching high accuracy (Li et al., 2017), which allows the program to incorporate the intricate associations among input parameters into algorithms (LeCun et al., 2015). In fact, due to the advances in computational capabilities, ANN has been widely optimized and used in medical informatics in recent years.

Previously, prehospital triage tools for detection of LVO were as simple as possible, in order to be easily memorized by emergency medical services personnel (Scheitz et al., 2017). However, the ignorance of various stroke-related parameters may reduce the accuracy of prediction. For example, patients with atrial fibrillation in the current study were more likely to have LVO, compared to those without. Our finding, that the ANN model containing more information, other than NIHSS items performed better than the model* only included NIHSS items, further confirms it. Actually, in the mobile internet era when users get the intuitive outputs by easily inputting measured parameters, leaving the complex underlying algorithm to the online calculation tool or local mobile application, the ability to discriminate is more important than simplicity of use in LVO prediction tools. With the help of ANN model, the number of input parameters can be large and it requires no exact mathematical relation between input parameters and outputs. Besides, biomarkers also play important roles in the diagnosis of diseases (Wang et al., 2017; Zou et al., 2018). With the improvement of electronic medical record networking system and the development of mobile laboratory, biomarkers which had been proved to be correlated with ischemic stroke, such as D-dimer, Cystatin C (Zou et al., 2017) and low-density Lipoprotein (Weng et al., 2018) can be added in the ANN model. Moreover, this is only a one-time computational cost that is incurred during training of the ANN model (Ferreri et al., 2010). Besides, these types of automatic diagnostic tools may perform better when they are integrated with more diagnostic information from the electronic medical record in the future.

Our study has some limitations. First, the model was derived and validated from a single cohort. Validation on an independent data set would be more compelling than cross-validation. Second, all patients were diagnosed with acute ischemic stroke and received reperfusion therapy. Consequently, sensitivity and specificity of the ANN model for LVO might differ in prehospital cohorts with suspected stroke that include stroke mimics and hemorrhagic strokes. Thus, we cannot rule out a selection bias, and studies performed in preclinical setting are necessary to generalize our results. Third, NIHSS evaluation was done at the time of admission to hospital by experienced doctors. But previous studies have demonstrated the elements from NIHSS scale used in ANN model have a good interobserver reproducibility by physicians and other health personnels (Goldstein and Samsa, 1997; Dewey et al., 1999). Fourth, the retrospective and observational design inherits potential for bias. Our results need to be confirmed prospectively in larger cohorts. Fifth, we didn’t do a statistical comparison between ANN model and conventional tools, since the ROC analysis of ANN model was done in 10 training datasets and 10 testing datasets, and the analysis of other conventional tools was done in the whole sample (600 patients).

In summary, we found that the ANN could be an effective tool to identify stroke patients with high likelihood of proximal LVO and it had higher accuracy than other prehospital prediction scales. Further prospective studies in the prehospital setting are needed to assess its practicability and accuracy.

## Author Contributions

ZC and RZ drafted and revised the manuscript, participated in study concept and design, conducted the statistical analyses, analyzed and interpreted the data. ML participated in study concept and design, data interpretation and made a major contribution in revising the manuscript. FX assisted in designing the ANN model and data analysis. XG, FS and MZ participated in the study design and made contribution in revising the manuscript.

## Conflict of Interest Statement

The authors declare that the research was conducted in the absence of any commercial or financial relationships that could be construed as a potential conflict of interest.

## Supplementary Material

FIGURE S1 Patient selection process.

TABLE S1 Comparison of baseline variables between LVO and non-LVO patients. LVO indicates large vessel occlusion.
