# Corrigendum: Novel Prehospital Prediction Model of Large Vessel Occlusion Using Artificial Neural Network

**DOI:** 10.3389/fnagi.2018.00222

**Published:** 2018-07-17

**Authors:** Zhicai Chen, Ruiting Zhang, Feizhou Xu, Xiaoxian Gong, Feina Shi, Meixia Zhang, Min Lou

**Affiliations:** ^1^Department of Neurology, The Second Affiliated Hospital of Zhejiang University, School of Medicine, Hangzhou, China; ^2^Department of Engineering, Microcloud Communication Technology, Hangzhou, China

**Keywords:** large vessel occlusion, artificial neural network, stroke, scale, NIHSS

There is an error in the Funding statement. The correct number for the Science Technology Department of Zhejiang Province is 2018C04011. The authors apologize for this error and state that this does not change the scientific conclusions of the article in any way.

The original article has been updated.

## Conflict of interest statement

The authors declare that the research was conducted in the absence of any commercial or financial relationships that could be construed as a potential conflict of interest.

