# Supplementary figures and images for: Novel Prehospital Prediction Model of Large Vessel Occlusion Using Artificial Neural Network
Source: Front Aging Neurosci. 2018 Jun 26;10:181. doi: 10.3389/fnagi.2018.00181 (PMC6028566; doi:10.3389/fnagi.2018.00181)

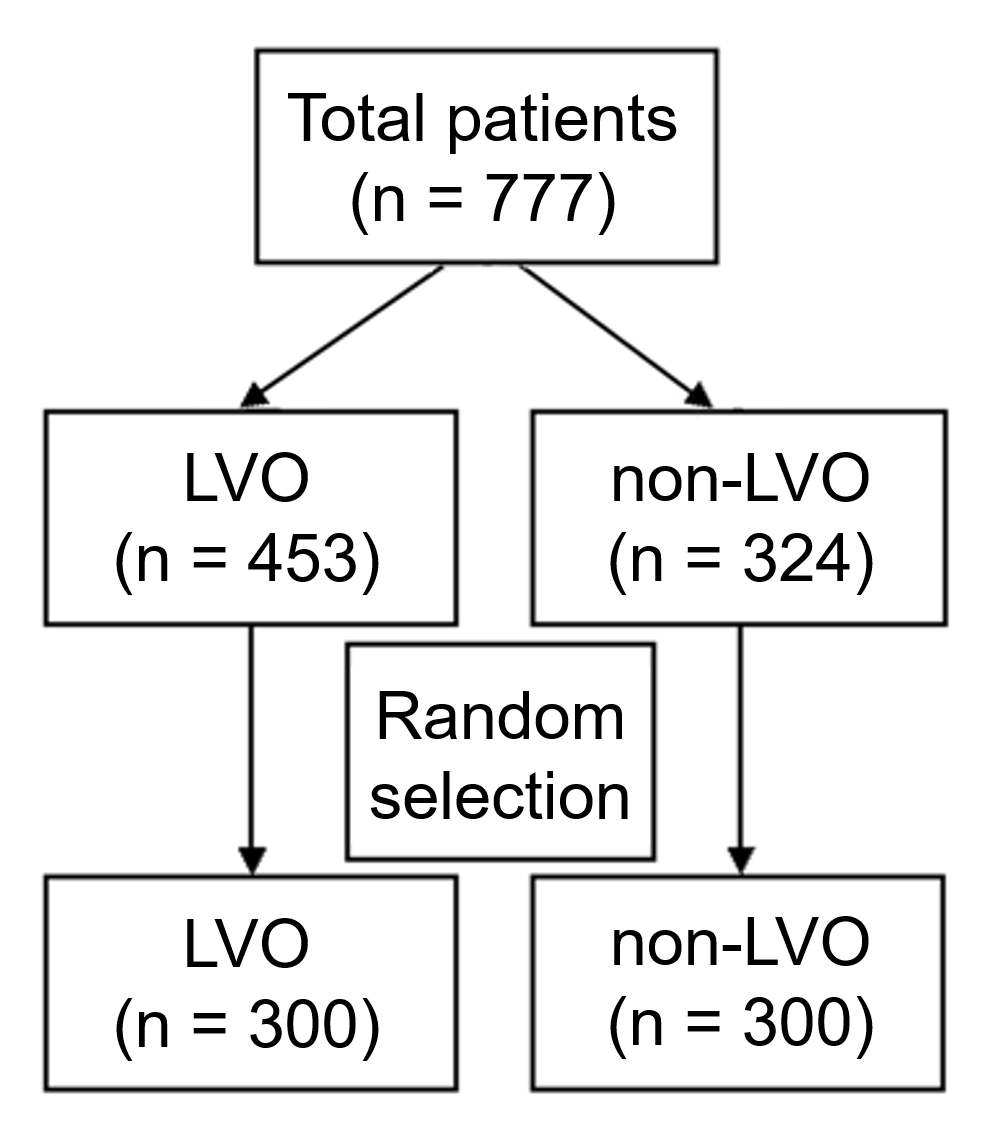

Supplement: FIGURE S1 — Patient selection process. [file Image_1.TIF]
