# Supplementary material for: Novel Prehospital Prediction Model of Large Vessel Occlusion Using Artificial Neural Network
Source: Front Aging Neurosci. 2018 Jun 26;10:181. doi: 10.3389/fnagi.2018.00181 (PMC6028566; doi:10.3389/fnagi.2018.00181)
Supplement: TABLE S1 — Comparison of baseline variables between LVO and non-LVO patients. LVO indicates large vessel occlusion. [file Table_1.DOCX]

**SUPPLEMENTAL MATERIAL**

**Additional Table 1.** Comparison of baseline variables between LVO and non-LVO patients

|  | LAO  (n = 300) | Non-LAO  (n = 300) | *p* value |
| --- | --- | --- | --- |
| Male (n,%) | 180 (60.0%) | 188 (62.7%) | 0.502 |
| Age (year) | 69±13 | 66±12 | < 0.001 |
| Prior antiplatelet therapy (n,%) | 43 (14.3%) | 55 (18.3%) | 0.224 |
| Smoking (n,%) | 103 (34.3%) | 112 (37.3%) | 0.444 |
| Hypertension (n,%) | 205 (68.3%) | 205 (68.3%) | 1.000 |
| Diabetes mellitus (n,%) | 54 (18.0%) | 64 (21.3%) | 0.304 |
| Hyperlipidemia (n,%) | 121 (40.3%) | 143 (47.7%) | 0.070 |
| History of stroke / TIA (n,%) | 45 (15.0%) | 53 (17.7%) | 0.377 |
| Atrial fibrillation (n,%) | 162 (54.0%) | 70 (23.3%) | < 0.001 |
| Coronary artery disease (n,%) | 34 (11.3%) | 30 (10.0%) | 0.597 |
| Hyperhomocystinemia (n,%) | 53 (17.7%) | 68 (22.7%) | 0.154 |
| Family history of cerebrovascular disease | 34 (11.3%) | 44 (14.7%) | 0.225 |
| NIHSS | 13 (8 - 17) | 6 (3 - 10) | < 0.001 |
| Level of Consciousness |  |  |  |
| A) LOC Responsiveness | 0 (0 - 1) | 0 (0 -0) | < 0.001 |
| B) LOC Questions | 1 (0 - 2) | 0 (0 - 1) | < 0.001 |
| C) LOC Commands | 0 (0 - 2) | 0 (0 - 0) | < 0.001 |
| Horizontal Eye Movement | 1 (0 - 2) | 0 (0 -0) | < 0.001 |
| Visual field test | 0 (0 - 0) | 0 (0 -0) | 0.528 |
| Facial Palsy | 1 (1 - 2) | 1 (0 - 1) | < 0.001 |
| Motor left Arm | 0 (0 - 4) | 0 (0 - 1) | < 0.001 |
| Motor right Arm | 0 (0 - 3) | 0 (0 - 1) | < 0.001 |
| Motor left Leg | 0 (0 - 3) | 0 (0 - 1) | < 0.001 |
| Motor right Leg | 0 (0 - 3) | 0 (0 - 1) | < 0.001 |
| Limb Ataxia | 0 (0 -0) | 0 (0 -0) | 0.130 |
| Sensory | 1 (0 -1) | 0 (0 -1) | < 0.001 |
| Language | 2 (0 -3) | 0 (0 -1) | < 0.001 |
| Speech | 1 (0 -2) | 1 (0 -1) | < 0.001 |
| Extinction and Inattention | 0 (0 -0) | 0 (0 -0) | 0.148 |

LVO indicates large vessel occlusion
